# Neurophysiological markers of network dysfunction in neurodegenerative diseases

**DOI:** 10.1016/j.nicl.2019.101706

**Published:** 2019-02-02

**Authors:** Roisin McMackin, Peter Bede, Niall Pender, Orla Hardiman, Bahman Nasseroleslami

**Affiliations:** a Academic Unit of Neurology, Trinity Biomedical Sciences Institute, 152-160 Pearse St., Trinity College Dublin, The University of Dublin, Ireland; b Computational Neuroimaging Group, Trinity Biomedical Sciences Institute, 152-160 Pearse St., Trinity College Dublin, The University of Dublin, Ireland; c Beaumont Hospital Dublin, Department of Psychology, Beaumont Road, Beaumont, Dublin 9, Ireland; d Beaumont Hospital Dublin, Department of Neurology, Beaumont Road, Beaumont, Dublin 9, Ireland

**Keywords:** MEG, TMS, EEG, Neurodegeneration, Network, Biomarker

## Abstract

There is strong clinical, imaging and pathological evidence that neurodegeneration is associated with altered brain connectivity. While functional imaging (fMRI) can detect resting and activated states of metabolic activity, its use is limited by poor temporal resolution, cost and confounding vascular parameters. By contrast, electrophysiological (e.g. EEG/MEG) recordings provide direct measures of neural activity with excellent temporal resolution, and source localization methodologies can address problems of spatial resolution, permitting measurement of functional activity of brain networks with a spatial resolution similar to that of fMRI. This opens an exciting therapeutic approach focussed on pharmacological and physiological modulation of brain network activity.

This review describes current neurophysiological approaches towards evaluating cortical network dysfunction in common neurodegenerative disorders. It explores how modern neurophysiologic tools can provide markers for diagnosis, prognosis, subcategorization and clinical trial outcome measures, and how modulation of brain networks can contribute to new therapeutic approaches.

## Highlights

• EEG/MEG/TMS provide measures of neuro-electric activity in the brain networks.
• Neuro-electrophysiological measurements have millisecond temporal resolutions.
• Source localisation of EEG/MEG can provide spatial resolution similar to fMRI.
• EEG/MEG/TMS objectively quantify network dysfunction in neurodegenerative diseases.
• Neuro-electrophysiology offers biomarkers and outcome measures for clinical trials.

## 1 Introduction

Modern clinical imaging, pathological (Yates, 2012) and genomic (Saura et al., 2015) data, support the evolving notion that neurodegenerative syndromes are best understood in terms of disrupted brain networking. Quantitative Magnetic Resonance Imaging (MRI) and Positron Emission Tomography (PET) provide compelling evidence of widespread network changes in neurodegenerations including Alzheimer's disease (AD) (Canter et al., 2016), Parkinson's disease (PD) (Gratwicke et al., 2015), amyotrophic lateral sclerosis (ALS) (Nasseroleslami et al., 2017) and frontotemporal dementia (FTD) (Bede et al., 2018). New therapeutic approaches based on network modulation are already in use for Parkinson's (Gratwicke et al., 2015) and Alzheimer's Disease (Canter et al., 2016).

Notwithstanding, characterizing changes in brain networking in a clinical setting remains a challenge. Structural MR imaging can show changes in grey and white matter integrity (Symms et al., 2004) and functional imaging (fMRI) detects resting and activated states of metabolic activity. Neither modality can directly measure neuronal activity, however. Furthermore, as fMRI measurements can be confounded by vascular pathology and are limited by the requirements of the technology (including the need for the patient to remain supine) (Glover, 2011), the use of fMRI is limited in the neurodegenerations. There remains an urgent and unmet need for user-friendly, non-invasive technologies that can rapidly and reliably detect network alteration with high temporal and spatial resolution.

Here we review the biology of non-invasive electrophysiology-based measurements and outline the current state of the art in measurement of network dysfunction in the neurodegenerations. We explore the future potential of emerging electrophysiology-based technologies in providing enhanced temporal resolution, and in using source localization that improves spatial resolution to complement structural and functional imaging.

## 2 Methods

### 2.1 Electroencephalography and magnetoencephalography

Quantitative EEG (qEEG) and magnetoencephalography (MEG) are increasingly recognized as useful non-invasive methods to measure cortical neurophysiological activity.

MEG and qEEG capture and digitise neuroelectromagnetic reflections of the synchronous generation of excitatory and inhibitory post-synaptic potentials in populations of underlying neurons. Both MEG and qEEG have excellent temporal but, until recently, limited spatial resolution. Several methods, collectively referred to as source localisation methods, have now been developed that enhance the spatial resolution of both EEG and MEG to that of using fMRI (Moeller et al., 2013). This now allows for visualisation of brain activity at low cost, with high levels of both spatial and temporal resolution.

The physiologic basis of MEG and EEG differ. MEG sensors measure the magnetic field generated by the electrical flows in neuronal populations while EEG sensors measure the simultaneously-generated perpendicular electric field that passes through the space between the activity source and sensors (da Silva, 2013). Due to volume conduction, EEG sensors also capture electrical currents propagated between the source and sensor in the conductive human head medium. This effect of volume conduction in EEG may make MEG a more reliable measure for deeper sources.

However, it must be noted that the potential advantage of MEG is reduced by the need for expensive superconductive systems (Wendel et al., 2009) that significantly increase costs, limiting MEG's day-to-day application in clinical settings.

**Fig. 1:**
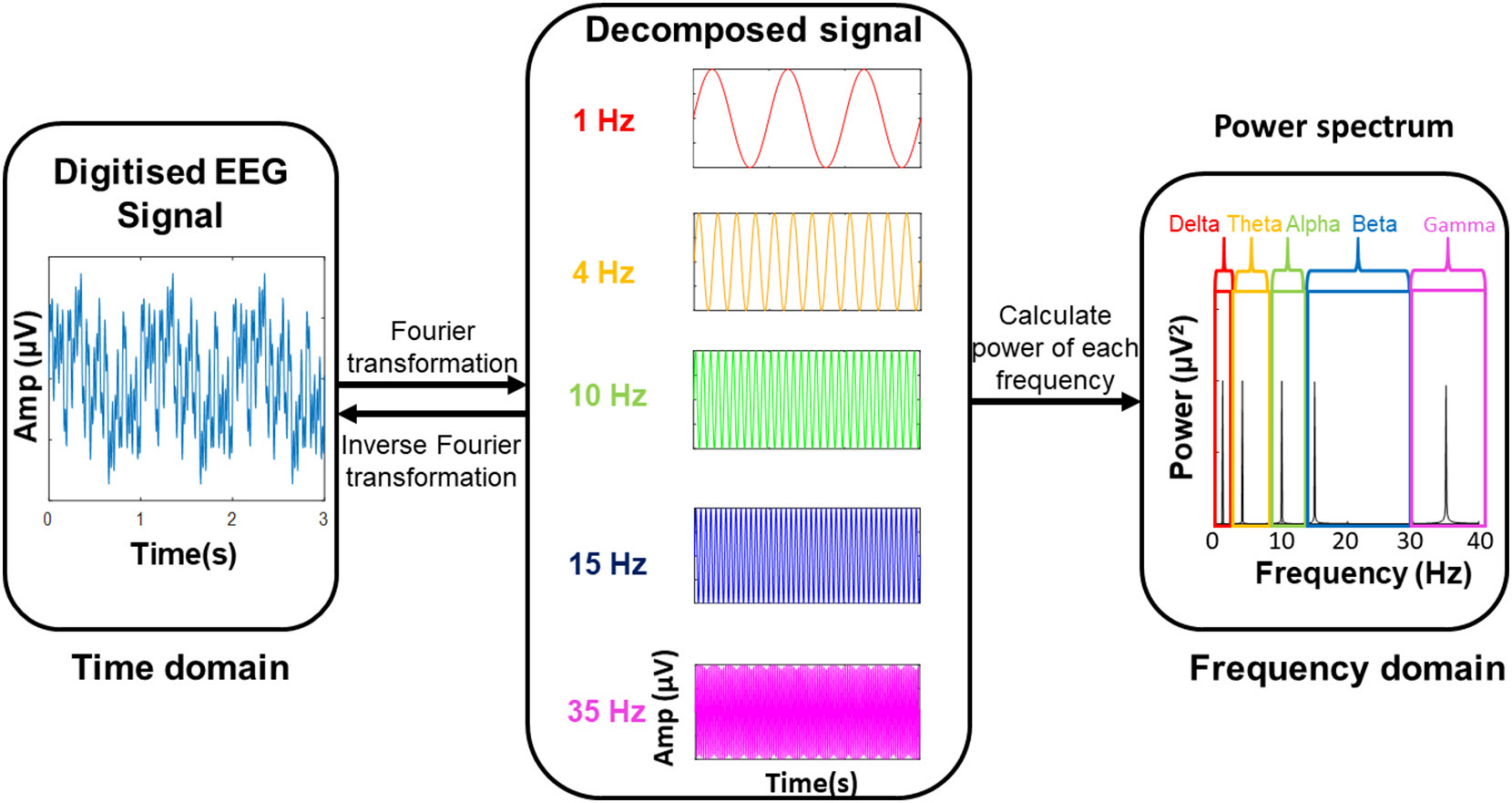
The transformation of a digitised EEG signal into a frequency power spectrum.

EEG and MEG both generate waveform data, where the x-axis represents time and the y-axis represents amplitude of electrical activity (Box 1). Quantitative M/EEG involves the digitisation of these signals and quantitative analysis of their characteristics (Fig. 1). These analyses can be performed in time and frequency domains. Time domain analysis is the study of how brain activity changes over time (Nuwer, 1997) (for example at what time the intensity of neural activity peaks when performing a cognitive or motor task). Frequency domain analysis involves the use of Fourier transformation to decompose the recording into a combination of waves of different frequencies.

Box 1 **Electrical and physiological characteristics defined in the context of EEG measurements.**

**Amplitude –** The size of the electrical charge in the cerebrospinal fluid produced by the summation of neuroelectric activity such as excitatory and inhibitory post synaptic potentials in cerebral cortical neurons, typically in microvolts (μV) (Cohen, 2014).

***Power –** A measure of the intensity of neuronal activity, proportional to the amplitude squared* (Cohen, 2014).

***Frequency –** The number of times a cycle of a wave repeats per unit time, measured in hertz (Hz)* (Cohen, 2014).

***Frequency bands –** Continuous ranges of frequencies for which measurements are grouped.*

***Oscillation –** Continuous, periodic neuronal activity, typically generated by feedback loops in neuronal networks* (Herrmann et al., 2016)

***Event-related potential (ERP) –** Electrical potential observed at the time that an event occurs, such as performing a motor or cognitive task or sensory stimulus* (Luck et al., 2000, p.)*.*

***Event-related (de)synchronisation (ERD/ERS) –** Relative decrease or increase in the intensity of oscillatory activity in a frequency band, caused by an event such as performing a motor or cognitive task or sensory stimulus* (Pfurtscheller and Lopes da Silva, 1999).

***Sensor-level** –Digitised M/EEG data analysed with respect to the position of the sensors on the scalp, providing poor spatial resolution.*

***Source-level** – Digitised M/EEG data analysed using source localisation methods to determine the location of contributing sources in the brain, providing spatial resolution comparable to fMRI* (Moeller et al., 2013).

Alt-text: Box 1

Typically, quantitative M/EEG signal frequencies are grouped into delta (0.5–3 Hz), theta (3–7 Hz), alpha (8–13 Hz), beta (14–30 Hz), and gamma (>30 Hz) frequency bands (Başar et al., 2001, p.). Oscillations in these different frequency bands have been attributed to different neuronal populations and brain activities (Herrmann et al., 2016) (Box 1). This allows for investigation of brain activity in terms of the power of oscillating network activity at different frequencies, referred to as spectral EEG (Kaiser, 2005). Synchronous or time-correlated oscillations in different brain areas can also be used to infer functional connectivity between them (Stam et al., 2007). The frequencies of these bands are generally negatively correlated to their amplitude (i.e. lower frequency M\EEG oscillations tend to have higher amplitude). Since amplitude is a reflection of the number of neurons contributing to a signal, lower frequency oscillations are attributed to synchronous activity of larger numbers of neurons (Pfurtscheller and Lopes da Silva, 1999).

**Fig. 2:**
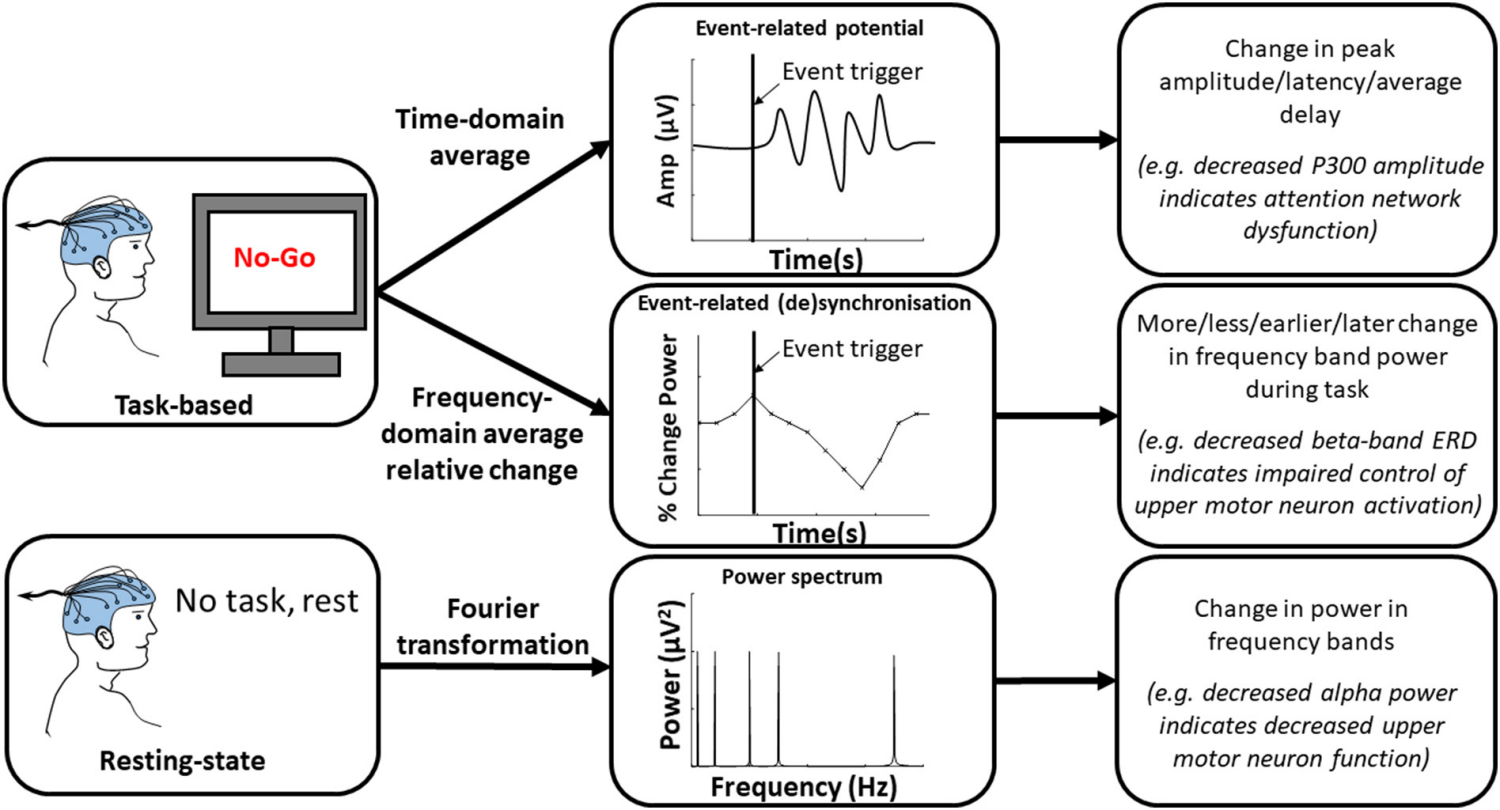
EEG signal processing avenues for resting-state and task-based paradigms, the quantitative measures obtained and sample interpretations in neurodegenerative disease.

These time and frequency domain network characteristics can be examined at rest (“resting-state”) to investigate the resting activity of the brain (Fig. 2). M/EEG measures can also be captured during tasks such as cognition, sensation or movement, to measure the activity of brain regions contributing to the generation of that function (Fig. 2) (Garrido et al., 2009; Shibasaki and Hallett, 2006). As tasks are underpinned by integration of various distinct neural networks, the corresponding neural signatures can be marked in the frequency domain, known as event-related (de)synchronisation (ERD/S), and/or the time domain, known as event-related potentials (ERPs) (Box 1). Source localisation methods can subsequently be applied to identify the origin of these of the network components and any changes to their performance in disease. Each of these approaches allows for the study of different aspects of neural network function and can be combined to provide a well-rounded insight into the effects of disease pathology on brain network function.

### 2.2 Transcranial magnetic stimulation (TMS)

TMS is the external application of a magnetic field to cortical neurons of interest, generating an electrical field around them. This electrical field will produce a charge across the membranes of the neurons in this area of the cortex, which will induce neuronal firing (e.g. the proliferation of an action potential along the axon) if of sufficient magnitude (Grunhaus et al., 2002). Using an electromagnetic coil placed on the scalp this magnetic field can be delivered in focal pulses to the cortical area of interest. Therefore TMS has the major advantage of providing a method to stimulate the cortex that is both non-invasive and focal, unlike transcranial electrical stimulation (Elder and Taylor, 2014).

**Fig. 3:**
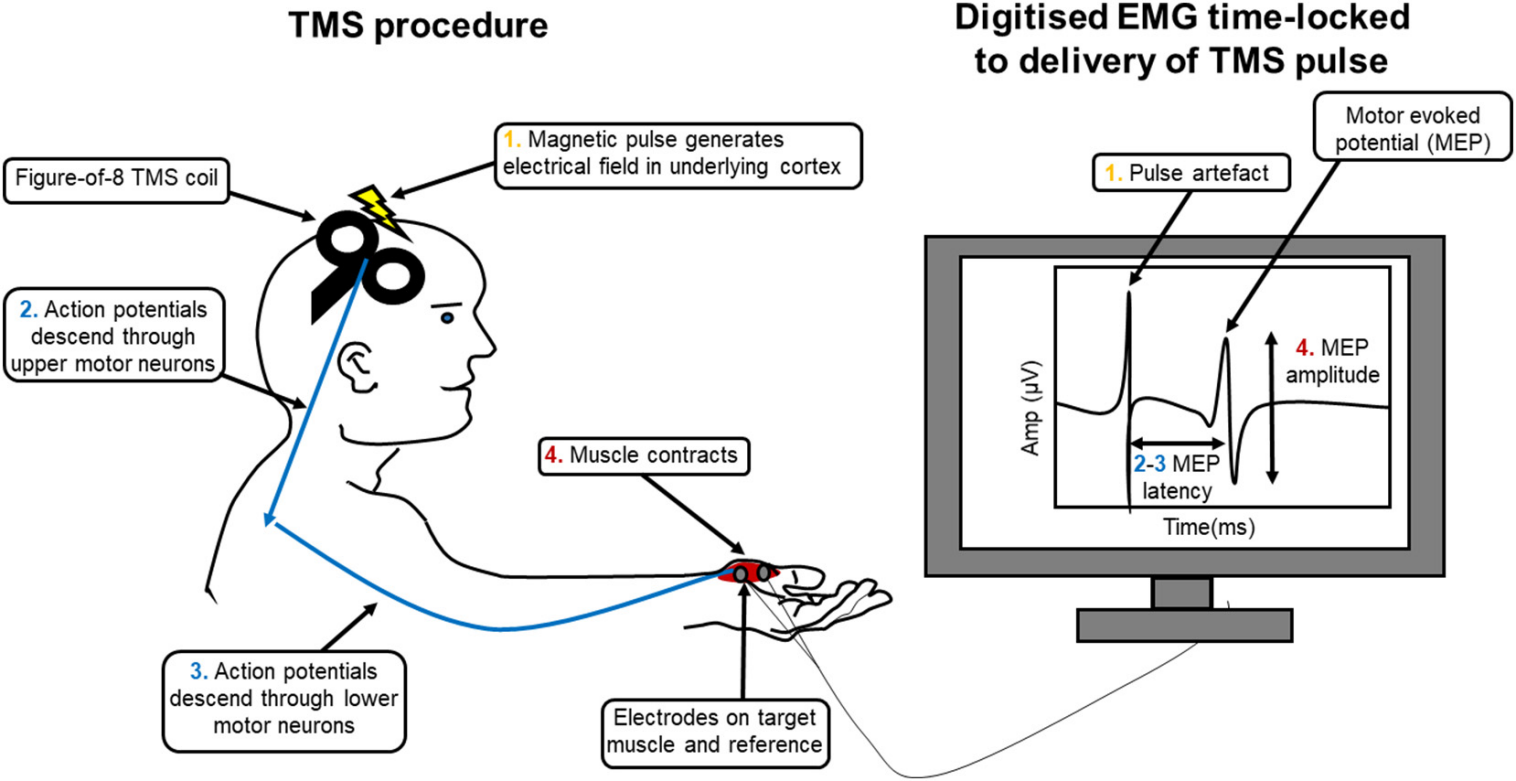
Schematic of a single-pulse TMS procedure and the quantitative characteristics of the resulting motor evoked potential.

TMS, coupled with surface electromyography (EMG) of muscles of interest can measure pyramidal tract function, anterior horn cell function and muscle activation (Fig. 3). By applying single stimulating pulses to the primary motor cortex, several commonly-used measures can be estimated, including: amplitude of the motor evoked potential (the EMG response to a stimulating pulse), the resting motor threshold (the minimum stimulation required to induce a standard motor evoked potential amplitude in 50% of electromyographic responses), cortical silent period (the period of interruption of voluntary muscle activity following stimulation of the corresponding motor cortical regions) and central motor conduction time (motor evoked potential latency less peripheral conduction time, measured by applying a TMS pulse at spinal level to the lower motor neurons innervating the target muscle) (Rossini et al., 2015).

Paired-pulse TMS provides the use of a conditioning stimulus (CS) at different intervals in advance of the test stimulus (TS) from either the same coil or a separate coil placed above another cortical region, usually over the opposite hemisphere. This can be used to study changes in inhibitory and excitatory circuits modulating motor cortical function. These measures include changes in short- and long-interval intracortical inhibition, intracortical facilitation, short- and long-interhemispheric inhibition and interhemispheric facilitation. Each of these measures is used to interrogate regulatory inputs to the corticospinal tract (Goss et al., 2012).

## 3 Network dysfunction in neurodegeneration

### 3.1 Resting state studies

“Resting state” EEG and MEG are used to explore brain activity and functional connectivity in the absence of specific tasks, although it must be acknowledged that the brain is continuously active with ongoing processing of both endogenous and exogenous information (Khanna et al., 2015). Neurodegenerative conditions exhibit changes in resting state that correlate with underlying pathogenic processes, and there is emerging evidence that resting state EEG has considerable discriminatory value in neurodegeneration.

In ALS, resting state EEG can identify changes in the sensorimotor cortex, as exemplified by the presence of decreased alpha-band power (Mai et al., 1998; Nasseroleslami et al., 2017; Santhosh et al., 2005). Alpha frequency oscillations over the sensorimotor cortex are attributed to the layer V pyramidal upper motor neurons (Jones et al., 2000), and as alpha power is known to decrease at movement onset (Pfurtscheller and Lopes da Silva, 1999) it is likely to represent the inactive state in these large cells. Loss of power in this band is therefore likely due to loss of cell bodies in this region, and possibly loss of inter-neuronal or thalamic control of the upper motor neurons at rest.

By contrast, broadband gamma power is increased over the motor cortex in PD, a finding that also differentiates PD from dystonia and essential tremor. This difference has been attributed to PD-related changes in the spiking of pyramidal cells (Crowell et al., 2012) and may aid in differential diagnosis. Increase in basal ganglia-cortical beta power is also consistently identified in PD (Giannicola et al., 2010; Jenkinson and Brown, 2011; Pollok et al., 2012). The pathological effect of such excessive oscillations has been established using deep brain stimulation, with 5–20 Hz stimulation, but not 30-50 Hz stimulation, exacerbating bradykinesia (Jenkinson and Brown, 2011).

Resting state EEG can also detect changes in brain connectivity. In ALS, resting state studies have identified increased connectivity throughout the cortex including increased median absolute coherence in theta and gamma band frequencies over prefrontal areas, accompanied by decreased gamma band synchrony for some prefrontal electrodes (Nasseroleslami et al., 2017). Cortical gamma band oscillations have been linked to higher cognitive functions such as intermodal selective attention and perception (Herrmann et al., 2016), providing a quantitative measure for detecting early cognitive impairment in ALS. In PD, decreased frontoparietal connectivity coherence in alpha band is also associated with early executive impairment (Teramoto et al., 2016), suggesting that deterioration of frontoparietal attention networks contributes to executive dysfunction in PD.

Numerous studies have highlighted the utility of combining such resting state EEG activity and connectivity measures for differential diagnosis of neurodegenerations, particularly the dementias (Nardone et al., 2018). For example, using temporal high beta, parietal theta and alpha and high beta power, a stepwise discrimination function can distinguish AD and FTD patients with 84.6% accuracy and is highly accurate in separating controls (100%) from FTD patients (84.6%) (Yener et al., 1996). With increase in computational power, this methodology has been enhanced, with training support vector machine classifiers using 25 EEG parameters capable of deciphering AD, PD, LBD and bvFTD with 100% specificity and sensitivity (Garn et al., 2017).

Such multidimensional biomarkers may also be enhanced by the addition of imaging and/or psychological task parameters to capture differences between broad, overlapping network pathologies. This has been demonstrated by logistic regression models combining cognitive task performance with delta and theta oscillatory activity which provide 93.3% accuracy when distinguishing AD from FTD (Lindau et al., 2003).

EEG measures can also quantify responses to drug therapies, for example in PD patients L-DOPA is found to induce widespread reduction in cortical delta and alpha activity, considered to reflect an excitatory effect of dopamine neuromodulation (Babiloni et al., 2019), in addition to suppressing elevated beta oscillations in correlation with motor improvement (Muthuraman et al., 2018). Such measures therefore have potential to provide objective, quantitative measures of drug effects on neurodegenerative pathology, enhancing the power of clinical trials. This potential has already been harnessed as a dose-finding pharmacodynamic biomarker in rodents, wherein dose-dependent increase in gamma band power in rats was used to estimate therapeutically relevant concentrations of a potential antidepressant drug in humans. This effect translated to similar increases in human resting-state EEG upon drug delivery (Sanacora et al., 2014).

Longitudinal resting-state M/EEG studies have been performed for a number of neurodegenerative conditions, but they are few in number. In AD, relative alpha and beta power is decreased, while relative theta and delta power increased longitudinally (Coben et al., 1985), with changes in relative theta power capable of distinguishing between different stages of dementia. This pattern is consistent across populations (Kwak, 2006; Verdoorn et al., 2011), demonstrating a global slowing in brain network signalling in AD.

Longitudinal increase in beta power has also been observed in PD, correlating with decline in Rey Auditory-Verbal Learning Test performance(Caviness et al., 2015), consistent with increasing delta power capturing progressive decline of specific cognitive networks. PD patients also show early impairment in brain network local efficiency as well as network decentralization which progress over time (Dubbelink et al., 2014).

In ALS a single longitudinal resting-state study has been reported revealing widespread, progressive increase in median coherence in theta and low gamma band frequencies (Nasseroleslami et al., 2017). This suggests that abnormal functional connectivity worsens throughout ALS pathology. Network activity may increase at disease onset and decline thereafter, and accordingly future studies will also require correlation with time from disease onset, and clinical stage of disease.

These studies demonstrate the ability of resting-state EEG to characterize and quantify neurodegenerations and their progression (see Table 1). In all cases, to attribute the recorded changes to specific networks, source localisation will be required. Moreover, future longitudinal studies will require extensive validation across large groups of well-phenotyped patients.

**Table 1:** Neurophysiological biomarkers for and therapies in neurodegeneration.

Technology	Method	Clinical application	Example		
			Disease	Biomarker/symptom	Reference
EEG/MEG	Resting state	Differential diagnosis	FTD, AD, PDD, DLB	Multiple	(Garn et al., 2017; Nardone et al., 2018; Yener et al., 1996)
		CT outcome measure	PD	Beta power	(Babiloni et al., 2019; Muthuraman et al., 2018)
TMS	Paired pulse	Diagnostic biomarker	ALS	SICI	(Menon et al., 2015; Vucic et al., 2008)
		CT outcome measure			NCT02450552, NCT02781454
rTMS	To leg area of motor cortex	Therapy	MS	Spasticity	(Mori et al., 2010, Mori et al., 2011
	To leg area of motor cortex		PD	Freezing of gait	(Chang et al., 2017; Kim et al., 2015) NCT02850159
	To dorsolateral prefrontal cortex or motor cortex			Refractory depression	(Lesenskyj et al., 2018)
	To dorsolateral prefrontal cortex		FTD, AD	Language, memory, executive function	(Antczak et al., 2018; Rutherford et al., 2015) NCT02621424
DBS	To basal ganglia	Therapy	PD	UPDRS score, mobility, activities of daily living, emotion, stigma, discomfort	(Deuschl et al., 2006)
	To nucleus basalis		AD	ADAS-cog	(Kuhn et al., 2015)

CT – Clinical trial. DLB – Dementia with Lewy Bodies, AD - Alzheimer's disease, PD – Parkinson's disease, FTD – Frontotemporal dementia, MMN – Mismatch Negativity, rTMS – Repetitive TMS, DBS – Deep brain stimulation, SICI – Short Interval Intra-Cortical Inhibition, UPDRS - Unified Parkinson's disease Rating Scale, ADAS-cog - Alzheimer's Disease Assessment Scale-Cog.

#### 3.1.1 Source localization studies

Source-level studies using quantitative EEG can correlate pathological neuroelectric signals with anatomic locations. For example, in AD increases in delta band activity are localised to orbitofrontal and temporal cortices, while frontotemporal dementia (FTD) patients differ, exhibiting decreases in low alpha band activity in these areas (Nishida et al., 2011). By contrast, reduced alpha activity in occipital sources and widespread increase in delta sources is revealed by source localisation in PD with and without cognitive impairment (Babiloni et al., 2019).

Source localisation can also be used to enhance the spatial resolution of connectivity measures. For example, localised lagged linear connectivity in alpha band has been found to discriminate AD, Dementia with Lewy Bodies and PD dementia from controls with areas under the ROC curves of 0.84, 0.78 and 0.75 respectively. Source localisation of EEG resting state connectivity in ALS patients has also revealed increased functional connectivity between the posterior parietal cortices (PPCs) and between the PPC and the motor cortex, dorsolateral, dorsomedial and ventrolateral prefrontal corticess. Source analysis also reveals increases in general connectivity of the anterior and posterior cingulate cortices, frontoinsular cortex, anterior insular cortex and dorsomedial and ventrolateral prefrontal cortices to other brain areas in ALS (Iyer et al., 2015). Source localised EEG measures therefore provide objective evidence that ALS and FTD have overlapping pathologies (Phukan et al., 2007), with cognitive networks disrupted in FTD, such as the frontoparietal attention networks (Zhou et al., 2010), also dysfunctioning in ALS, while central and parietal activity known to be abnormal in ALS (Nasseroleslami et al., 2017), is found to distinguish FTD from AD (Nishida et al., 2011).

### 3.2 Activation studies

#### 3.2.1 Event-related M/EEG

Network performance can also be quantified by measuring frequency or time domain characteristics of M/EEG signals generated by the performance of motor (Shibasaki and Hallett, 2006), sensory (Momma et al., 1987) or cognitive (Luck et al., 2000) tasks designed to activate target neural networks.

##### 3.2.1.1 Motor tasks

M/EEG can provide quantitative measures of motor network performance during movement. Movements are preceded by decrease in alpha and beta band oscillation power in the primary motor cortex. This is referred to as event-related desynchronisation (ERD). ERD is interpreted as an electrophysiological correlate of increasing activity in cortical areas involved in the movement (Pfurtscheller and Lopes da Silva, 1999). ERD is therefore used to quantitatively measure motor cortex dysfunction in disease. For example, in multiple sclerosis (MS), latency of ERD correlates with structural MRI T1 lesion volume and T2 lesion load (Leocani et al., 2005), while in PD, ERD begins closer to movement onset (Defebvre et al., 1994), particularly in the affected hemisphere (Defebvre et al., 1996). This difference is partially corrected by L-DOPA (Defebvre et al., 1998). By contrast, ERD is conserved in the upper motor neuron syndrome of Primary Lateral Sclerosis, despite the presence of decreased amplitude in movement-related potentials (Bai et al., 2006), suggesting that changes in ERD may quantify dysfunction of cells that regulate the primary motor cortex or non-upper motor neuron cells that receive thalamo-cortical input.

Following ERD, in the first second after movement ends, increased beta-band oscillations are recorded in the primary motor cortex, most prominent over the contralateral sensorimotor cortex. This is referred to as beta event related synchronisation (ERS) and is attributed to a shift of the primary motor cortex from activation back to an inactive state (Pfurtscheller and Lopes da Silva, 1999). Change in post-movement ERS has also been documented in MS, PD and ALS, providing additional quantitative measurement of motor cortex dysfunction. In MS, the latency of the ERS peak is significantly later and correlates to longer information processing speeds (Barratt et al., 2017), while in both ALS (Riva et al., 2012) and PD (Diez et al., 1999) ERS is reduced, even during dopaminergic treatment (Pfurtscheller et al., 1998). In ALS, negative correlations between ERS and measures of structural (subcortical frontal apparent diffusion coefficient) and functional (MEP to compound muscle action potential ratio) corticospinal tract integrity have also been reported (Riva et al., 2012). Increase in ERS may therefore represent a measure of impaired inhibition or excess activity of upper motor neurons.

The time domain characteristics of M/EEG can provide additional neurophysiological correlates of motor tasks, known as movement related potentials (MRPs) (Luck et al., 2000). Two major MRPs are elicited during motor planning. These are the Bereitschaftspotential (BP) (Shibasaki and Hallett, 2006) and the contingent negative variation (Rockstroh et al., 1993), providing measures of contributing motor preparatory and planning networks' function.

Source localisation has attributed the early BP to the supplementary motor area and premotor cortex bilaterally, followed by activity in the contralateral premotor and primary motor cortices (Shibasaki and Hallett, 2006). In PD, BP peak amplitude is not affected in patients compared with controls, but the early part of the of the waveform is attenuated (Dick et al., 1989). Decrease in peak amplitude does, however, correlate with increasing disease severity (Patil et al., 2017). This may reflect inadequate activation of the supplementary motor area by the basal ganglia (Dick et al., 1989) or supplementary motor area pathology in PD. Comparable findings in ALS, wherein BP amplitude is inversely correlated with spasticity (Westphal et al., 1998), demonstrate an overlap in the network pathology of these two neurodegenerations in the basal ganglia and/or the supplementary motor area. Such clinical correlation also points to a utility of these measures as prognostic biomarkers.

The contingent negative variation (CNV) has been localised in part to the premotor cortex and supplementary motor area (Hultin et al., 1996); however, CNV also represents prefrontal network activity in the orbitofrontal, mesial and dorsolateral prefrontal cortices, unlike the BP (Ikeda et al., 1996), therefore capturing additional motor preparatory network components. Mean amplitude of CNV is increased in ALS (Hanagasi et al., 2002), decreased in PD (Pulvermüller et al., 1996) and MS (Praamstra et al., 1996; Uysal et al., 2014) and unaffected in Alzheimer's disease (AD) (van Deursen et al., 2009). The discrepancy between ALS-related BP and CNV abnormalities suggests that prefrontal network decline makes an important contribution to changes in this ERP, consistent with the now-well established cognitive component of ALS pathology (Phukan et al., 2007). Furthermore, decrease in CNV amplitude over the parietal cortex in MS correlates with neuropsychological test performance (Uysal et al., 2014). This suggests that CNV also captures parietal network components pertaining to movement preparation and planning.

Localisation analyses have yet to identify the source(s) causing the disease-related abnormalities in MRPs. Such analyses are likely to reveal which cognitive and motor network components contribute to MRP changes in each of these neurodegenerations, highlighting any network overlap and potentially providing distinguishing biomarkers.

##### 3.2.1.2 Sensory tasks

Somatosensory ERPs, commonly referred to as SEP or SSEP, can provide information about the involvement of primary somatosensory cortex and its inputs in neurodegenerative diseases. For example, dysfunction of thalamocortical neurons of the ascending somatosensory tracts can be shown in ALS and HD. N20, an ERP generated by median nerve stimulation, is attributed to the initial primary somatosensory cortex in somatosensation (Banoub et al., 2003). N20 has increased latency in HD (Abbruzzese et al., 1990) and ALS (Zhang et al., 2014) patients, indicating pathological delay in transmission of stimuli to the cortex. In ALS, N20 latency increase occurs in the presence of normal peripheral conduction time, while in HD P15 latency (attributed to the brainstem (Momma et al., 1987) is normal (Josiassen et al., 1982), indicating that these impairments represent dysfunction of thalamocortical neurons of the ascending somatosensory tracts in ALS and HD pathology. Decrease in N20 amplitude also correlates to disease duration in ALS (Iglesias et al., 2015), which may reflect spread of pathology from the motor cortex to the primary somatosensory with disease progression.

##### 3.2.1.3 Cognitive tasks

A variety of different cognitive ERPs and ERP subcomponents have been used to objectively assess performance of different cognitive tasks in neurodegeneration, including P3 and mismatch negativity.

P3 is a positive peak seen in the average ERP 200-500 ms after an infrequent ‘deviant’ stimulus is delivered in a train of attended ‘standard’ stimuli, known as an oddball paradigm. It has been associated with inhibition of cortical networks to facilitate delivery of attention stimuli in the aftermath of an alerting signal (Polich, 2007), and therefore can be used to quantify attention network impairment in neurodegenerative disease. For example, as P3 latency is longer for more complex stimulus evaluation and decision making tasks (Polich, 2007), P3 latency is used to test the speed of attentional processes.

P3 latency is increased in MCI (Lai et al., 2010), AD (Pedroso et al., 2012), ALS (Gil et al., 1995) and PD (Tokic et al., 2016) and is predicted by lesion load in MS (Kimiskidis et al., 2016). P3 has been shown to be delayed or absent in 100% of a small group of cognitively impaired ALS patients (Portet et al., 2001) and is inversely correlated to performance in cognitive tasks globally, as well as specifically for language and attention in AD (Lee et al., 2013).

Mismatch negativity (MMN, also referred to as N2a) is another cognitive ERP generated by oddball paradigms, however unlike P3, MMN has the advantage that it does not require active patient participation. MMN is a negative peak at approximately 200 ms post-stimulus seen when the average ERP following a standard stimulus is subtracted from the average response to deviant stimuli. MMN is a physiological measure of working sensory memory, involuntary attention switching and sensory accuracy, therefore capturing both cognitive and sensory networks (Garrido et al., 2009).

MMN shows increased average delay correlating to response-inhibition task performance in ALS (Iyer et al., 2017), while in both PD and MS MMN is reduced in cognitively impaired patients compared to those without cognitive impairment (Brønnick et al., 2010; Jung et al., 2006). Reduced MMN amplitude is also reported in MCI and AD as reviewed by Horvath et al. (2018). Such cognitive correlations to MMN impairment point to the potential of MMN an additional quantitative measure of network dysfunction in neurodegeneration.

Few longitudinal studies of change in cognitive ERPs have been published, although in AD the P3 latency has repeatedly been shown to increase over time (Ball et al., 1989), with latency increase being more substantial in those with greater cognitive decline (St Clair et al., 1988). In ALS, correlation studies have found that P3 amplitude is related to disease duration (Volpato et al., 2010) and that P3a latency correlates to months from disease onset and symptoms severity (Raggi et al., 2008), consistent with progressive network decline with disease progression.

Source analysis of MMN and P3 can distinguish different degenerations with similar sensor-level ERP changes and provide more information about neurodegenerative pathology. To date however, few studies have utilised source analysis to determine the exact location of the networks producing such abnormalities, and the spatial resolution of existing findings remains to be definitively established.

#### 3.2.2 Transcranial magnetic stimulation

TMS has been established for three decades as a useful tool that interrogates cortical and potentially subcortical motor networks (Rossini et al., 2015). TMS can interrogate motor cortical excitability and has demonstrated that hyperexcitability is a feature of feature of ALS, PD and HD, although the excitable characteristics of these conditions differ (discussed below).

Resting motor threshold (RMT), a TMS-based measure of upper motor neuron excitability, is decreased in ALS (Grieve et al., 2015; Vucic et al., 2008) and AD (Liepert et al., 2001) but not in PD (Ni et al., 2013) or HD (Abbruzzese et al., 1997). Conversely, PD patients show greater motor evoked potential (MEP) amplitudes at low stimulus intensity (Leon-Sarmiento et al., 2013) and an inverse correlation between motor impairment and RMT (Park et al., 2016).

TMS can also interrogate the function of intracortical circuits regulating the corticospinal tract. SICI is a measure of the increase in muscle response to cortical magnetic stimulation due to a preceding conditioning stimulus from the same coil and is a measure of inhibitory interneuron function (Ziemann et al., 1996). Huntington's disease (HD), AD, PD and ALS each exhibit reduced short intracortical inhibition (SICI) (Abbruzzese et al., 1997; Grieve et al., 2015; Liepert et al., 2001; Ni et al., 2013; Pierantozzi et al., 2002; Vucic et al., 2008). This suggests that reduced inhibitory input to upper motor neurons contributes to corticospinal tract hyperexcitability. SICI may also capture dysfunction of dopaminergic circuitry. Dopaminergic drugs can increase SICI, while anti-dopaminergic drugs decrease SICI (Ziemann et al., 2015). Furthermore, in PD, dopaminergic drugs and BG deep brain stimulation can partially rectify reduced SICI (Ni et al., 2013; Pierantozzi et al., 2002). In AD, SICI decrease correlates with cognitive decline, and can be partially counteracted by donepezil (Liepert et al., 2001), also suggesting some cholinergic input to the SICI-generating circuitry.

Intracortical facilitation (ICF) is the increase in muscle response to cortical magnetic stimulation due to a preceding conditioning stimulus from the same coil. The interstimulus interval values giving rise to ICF are higher than those for SICI. ICF is increased in HD (Abbruzzese et al., 1997), ALS (Grieve et al., 2015; Vucic et al., 2008) and PD (Ni et al., 2013). The circuitry underlying ICF is relatively poorly understood, although novel investigation using the threshold tracking method indicates that short and long ICF measures of different circuitry exist, which differ in underlying circuitry from each other and that of SICI (Van den Bos et al., 2018). Pharmacological studies suggest ICF also involves GABAergic and dopaminergic circuitry (Ziemann et al., 2015). Consistent with this hypothesis, the ICF increase in PD can be partially counteracted by dopaminergic treatment (Ni et al., 2013).

Both increased ICF and decreased/absent SICI have been reported in three pre-symptomatic SOD-1 mutant carriers who later developed ALS (Vucic et al., 2008), while increased RMT has been found in preclinical and very early HD (Schippling et al., 2009).

These observations point to the potential utility of TMS-based biomarkers of early neurodegeneration (see Table 1).

Longitudinal TMS studies in ALS show decreases in MEP amplitude and increases in RMT (Floyd et al., 2009) and cortical silent period (Mills, 2003) with progression of the disease. SICI also correlates with measures of disease progression (compound muscle action potential, strength-duration time constant and neurophysiologic index) (Vucic and Kiernan, 2006) in TT-TMS studies. This is consistent with early excess excitation which later declines with degeneration of the motor system, leading to loss of function. In keeping with this hypothesis, RMT is decreased in patients who do not exhibit a weakness, wasting or upper motor neuron symptoms, but increased in those with lower and upper motor neuron symptoms (Mills and Nithi, 1997).

## 4 Therapeutic approaches using network modulation

### 4.1 Electrical and magnetic stimulation

Given the extensive literature of network dysfunction across the neurodegenerations, the neurophysiological modulation of these abnormalities presents a potential therapeutic target for these disorders (see Table 1). In addition to the utility of deep brain stimulation in artificially maintaining basal ganglia function in PD, it is now known to have a separate therapeutic effect on the disease, improving motor function and emotional well-being compared to medication alone (Deuschl et al., 2006). In a small study of AD patients stimulation of the nucleus basalis of Meynert stabilises or improves cognition over a year (Kuhn et al., 2015), illustrating the potential utility of deep brain stimulation in other brain network disorders.

TMS can also be used to deliver trains of magnetic stimuli to any part of the cortex, typically at least once per second, in order to alter network activity. This is known as repetitive TMS (rTMS) and has recently been approved as a therapy for treatment-resistant depression (George et al., 2013). RTMS has now been found to have therapeutic effects in a number of neurodegenerative diseases. Such effects include reduction of spasticity in MS (Mori et al., 2010, Mori et al., 2011), improved cognition and functionality in FTD (Antczak et al., 2018), improved cognition and reduced cognitive decline in AD (Rutherford et al., 2015) and reduced freezing of gait in PD (Kim et al., 2015). Furthermore, six out of seven studies investigating the effects of rTMS on refractory depression in PD identified significant improvement (Lesenskyj et al., 2018).

Some such effects are already being brought towards clinical practice. For example, rTMS is currently being investigated as a network modulating therapy for dementia in MCI or AD (NCT02621424) and spasticity in MS (NCT02747914, NCT01106365). A completed trial of rTMS in PD (NCT03219892) has also identified a significant therapeutic effect on freezing of gait as well as ambulatory and motor function (Chang et al., 2017).

### 4.2 Pharmacological network modulation

Pharmacological intervention to rectify network dysfunction is being investigated in a number of neurodegenerations. In addition to the correction of neurophysiological measures by existing drug therapies (Defebvre et al., 1998; Liepert et al., 2001; Ni et al., 2013), novel neurotherapeutics are being investigated on the basis of their network modulating properties. In ALS, a recent retigabine trial has used decrease in SICI as a recruitment criterion (NCT02450552) while a trial of mexiletine (NCT02781454) is now using change in RMT and SICI as primary and secondary outcome measures respectively. Resting-state EEG was also utilised as a secondary outcome measure in testing the nutritional aid Souvenaid as a therapy in AD, with change in delta band functional connectivity showing improved trajectory (Scheltens et al., 2012).

A combination of multimodal evoked potentials was also used an outcome measure in a phase III trial (NCT01765361) of the recently approved drug ocrelizumab for MS.

These early studies point to a move towards therapies based on modulation of network dysfunction, allowing for earlier, and possibly presymptomatic intervention based on early changes in physiological measures.

## 5 Conclusion

Neurophysiological recording and neuro-electric/−magnetic signal analysis can characterize patterned changes of network function in neurodegeneration, opening up opportunities for novel biomarkers of disease progression. The attractive properties of neurophysiological measurements have often been overlooked in the past. The development of focal TMS and source localisation of M/EEG signals can now provide direct measurements of network activity with high spatiotemporal resolution. These new developments provide additional opportunities for neurophysiology-based signal analysis as an additional investigational tool in neurodegeneration.

Directly quantifying network activity can be used to objectively identify neurodegeneration without relying on subjectively-measured symptoms which manifest from network dysfunction. This can allow for earlier and potentially presymptomatic intervention, providing greater probability of therapeutic success. Such measures are already being harnessed in clinical trials, however their full potential as outcome measures is still underexploited.

Neuroelectric signalling studies have already sufficiently demonstrated the importance of network dysfunction in neurodegeneration to drive development of network modulating stimuli and drugs as the therapeutic options and suggests that other pharmacologic agents that act to modulate network dysfunction are likely to be of therapeutic benefit. Additional studies are now required to fully exploit the potential of M/EEG and TMS across the range of neurodegenerations, including additional processing and source localization that can discriminate different disease subtypes.
